# Blood cultures to define neonatal early-onset sepsis: why not enough for clinical care?

**DOI:** 10.1038/s41390-026-04883-y

**Published:** 2026-03-07

**Authors:** Eleanor J. Molloy, Karen M. Puopolo, Richard Polin

**Affiliations:** 1 Discipline of Paediatrics, Trinity College Dublin, the University of Dublin, Dublin, Ireland; 2 Trinity Translational Medicine Institute (TTMI), St James Hospital, Dublin, Ireland; 3 Trinity Research in Childhood Centre (TRiCC), Dublin, Ireland; 4 Neonatology, Children’s Health Ireland (CHI) at Crumlin & Tallaght, Dublin, Ireland; 5 Neurodisability, Children’s Health Ireland (CHI) at Crumlin & Tallaght, Dublin, Ireland; 6 Paediatrics, Coombe Women’s and Infant’s University Hospital, Dublin, Ireland; 7 Division of Neonatology, Children’s Hospital of Philadelphia, Philadelphia, PA USA; 8 Department of Pediatrics, University of Pennsylvania Perelman School of Medicine, Philadelphia, PA USA; 9 College of Physicians and Surgeons, New York, NY USA; 10 Children’s Hospital of New York, New York, NY USA; 11 Columbia University, New York, NY USA

Commentary on: Dimopoulou V, Klingenberg C, Navér L, Nordberg V, Berardi A, El Helou S, Fusch G, Bliss JM, Lehnick D, Guerina N, Seliga-Siwecka J, Maton P, Lagae D, Mari J, Janota J, Agyeman PKA, Pfister R, Latorre G, Maffei G, Laforgia N, Mózes E, Størdal K, Strunk T, Stocker M, Giannoni E; AENEAS Study Group. Antibiotic exposure for culture-negative early-onset sepsis in late-preterm and term newborns: an international study. Pediatr Res. 2024 Sep 17. doi: 10.1038/s41390-024-03532-6. Epub ahead of print. PMID: 39289592.

## Neonatal sepsis definitions and microbiology

Although sepsis is a common reason for admission to the adult, pediatric and neonatal intensive care units, there is wide variation in definitions and diagnostic tools.^1^ The Sepsis-3 consortium developed a sepsis definition for adult patients as follows: “Sepsis is defined as life-threatening organ dysfunction caused by a dysregulated host response to infection”.^2^ Although culture results are useful to guide targeted antimicrobial therapies, positive microbiological findings are not an essential component of this definition. Instead, the Sepsis 3 definition relies on the Sequential Organ Failure Assessment (SOFA) score to define organ dysfunction in the setting of suspected infection.^3^ The Society of Critical Care Medicine Pediatric Sepsis Definition Task Force developed the PHOENIX diagnostic criteria for pediatric sepsis using international datasets and a systematic evaluation of models to predict in-hospital mortality among infants and children with suspected sepsis.^4^ Similar to Sepsis-3, “suspected sepsis” was defined by microbiological testing and receipt of systemic antimicrobial medications. The PHOENIX score utilized quantitative measures of respiratory, cardiovascular, coagulation and neurologic dysfunction to create a numeric score that predicts mortality among children in both low/middle income settings and high income settings. Ultimately, the PHOENIX group consensus aligned with the Sepsis-3 definition of sepsis, focusing on organ dysfunction: sepsis was defined as a PHOENIX score ≥2. Notably, the laboratory values utilized in the PHOENIX sepsis score are lactate, platelet count and measures of coagulation, and do not include inflammatory markers such as C-reactive protein or procalcitonin.

In contrast, there is no consensus endorsing a functional definition of neonatal sepsis. Infection occurring shortly after birth, referred to as neonatal early-onset sepsis (EOS) is most commonly defined by bacteremia (and more rarely meningitis) and not defined by organ dysfunction criteria. The primary reason for relying on microbiologic results is that organ dysfunction itself—in particular, cardiac and respiratory dysfunction—is common in the immediate neonatal period. Organ dysfunction may be attributed to physiologic cardiorespiratory transition among term infants and to organ system immaturity among preterm infants, as well as to birth asphyxia among all newborns. Further, efforts are often made to identify bacteremia at birth before the development of severe organ dysfunction, with the goal of isolating pathogens in blood culture prior to the occurrence of severe illness in the newborn. Neonatal late-onset sepsis (LOS, pccurring more than 3–7 days after birth) may be more amenable to a combination of microbiologic and organ dysfunction criteria. However, LOS is complicated by its application to both otherwise healthy infants who present with illness after birth hospital discharge, and to infants continuously hospitalized from birth due to preterm gestation or birth complicated by congenital medical or surgical conditions. Frameworks such as the PHOENIX score can be applied to infants after birth hospital discharge. Nonetheless, for both EOS and LOS, a microbiologic definition of infection allows for consistency within international benchmarking across populations - although such a definition does not inform clinical management beyond antibiotic administration.

## Antibiotic use in term and near-term infants

The recent study by Dimopoulou et al. from the AENEAS group highlights the challenges faced by clinicians in the diagnosis of EOS in term and near-term infants.^5^ This follows on their work demonstrating wide variation in antibiotic exposure among near-term and term infants.^6^ The current study retrospectively analysed data across 11 countries for 757,979 infants born ≥34 weeks’ gestation, focusing on the 21,703 (2.9%) who received antibiotics in the first week after birth. The study addressed outcomes among infants with and without confirmed bacteremia treated for <5 days or ≥5 days with antibiotics. Overall, infants with sterile blood cultures accounted for 21-fold more antibiotic exposure compared to those with culture-confirmed infection. Significant center-level variation was observed in the rate of antibiotics administered for culture-negative conditions: most notably, a 49-fold difference was seen comparing different centers with respect to the administration of antibiotics for <5 days when cultures were sterile. Mortality among infants administered antibiotics for ≥5 days with sterile cultures was attributed to causes such as congenital anomalies and perinatal asphyxia, whereas all the deaths associated with culture-confirmed infection were attributed to sepsis. The study found a decrease in antibiotic use in many networks over time, potentially reflecting antimicrobial stewardship efforts to rationalize antibiotic administration. The AENAES authors conclude that antimicrobial stewardship programs should focus on shortening antibiotic treatment for culture-negative cases. In demonstrating international disparities in sepsis management and antibiotic use, this study highlights the essential challenge of making progress in treating “culture-negative” sepsis in the newborn: how can we improve care for a condition if we cannot agree on now to define and diagnosis it?

## Challenge in neonatal sepsis diagnosis

If clinical sepsis is defined by dysregulated organ function in the setting of suspected infection, then birth is a terrible time to diagnose clinical sepsis. Infection is frequently suspected due to the known vulnerability of newborns to specific bacterial pathogens, and neonatal physiologic transition among term infants and organ immaturity among preterm infants drain the specificity from the occurrence of organ dysfunction. Birth is also characterized by the initial constitution of the human microbiome, and the transition of the newborn from an immune system based in innate immunity and passively-derived maternal antibody to one based on acquired immunity.^7,8^ Fundamental immaturity of the immune system and dysregulated immune response variably characterize the newborn response to infection. Further, the process of birth itself mediates changes in immune cell expression and production of inflammatory molecules.^9,10^ Together these factors result in clinical practice characterized by a low threshold to suspect infection and administer empiric antibiotics shortly after birth. To optimize such practices, multiple factors have been studied for utility in EOS risk assessment, including factors epidemiologically associated with sepsis risk such as birth socioeconomic setting, gestational age at birth, occurrence of preterm labor, preterm rupture of membranes (ROM), duration of ROM and administration of intrapartum antibiotics, while other factors are used as markers of developing infection, such as maternal intrapartum fever and other clinical signs of intraamniotic infection, and measures of fetal well-being such as tachycardia and heart rate variability.^11–13^ The connection between maternal and fetal/neonatal infection is illustrated by the fact that maternal bacteremia is associated with both culture-confirmed EOS and the diagnosis of culture-negative sepsis.^14^ Multivariate assessment of risk factors known at the time of birth combined with newborn clinical findings has been used to assess EOS risk among term and late preterm infants and determine the need for empiric antibiotics.^15–17^ Risk categorization focused on both the indication for preterm birth and the mode and details of delivery (vaginal versus caesarean, presence or absence of labor) can be used to determine the need for EOS evaluation and empiric therapy among preterm infants.^18–21^ Clinical tools such as the neonatal sequential organ dysfunction score (nSOFA) can predict morbidity and mortality in culture-confirmed EOS in the NICU.^22,23^ Details on the clinical condition of newborns and risk assessment strategies used to initiate antibiotic therapy were not provided in the AENEAS group study, a significant limitation in understanding the observed variation in diagnosis and practice.^5^ Additionally, the study provided no data on outcomes other than death, nor on recurrent infection or hospital readmission, limiting the opportunity to assess possible secondary benefits – or harms – attributable to antibiotic administration in the absence of confirmed bacteremia.

## Neonatal blood culture performance

Although the facts that microbiologic results were sought and empiric antibiotics administered served to define the target population for both the Sepsis-3 and PHOENIX studies, in neither study were microbiology results are an essential component of the final sepsis definition.^2,4^ Bacteremia is only detected in 50% of adults with clinical signs of sepsis and this percentage is lower if there is ongoing antibiotic therapy.^24–28^ In adults, cultures of specific organ sites (e.g., pulmonary) do not correlate well with blood culture results and may result in unnecessary or prolonged antibiotic treatment.^25–28^ In adult and pediatric sepsis, organ-specific infections such as urinary tract infection, and non-bacterial infections such as influenza may serve as the inciting event to generate sepsis. In contrast, a far narrower spectrum of pathogens and conditions afflict newborns. This reality is the basis for defining neonatal EOS by the occurrence of bacteremia or meningitis, yet the utility of blood culture in detecting neonatal bacteremia has been debated for decades. One study conducted in the 1990s found that 1 mL of blood in neonatal blood culture may still be insufficient to detect bacteremia in neonates with very low colony counts <4 CFU/mL.^29^ More contemporary blood culture systems can detect bacteremia at a level of 1–10 CFU/mL with a 1 mL minimum blood inoculate.^30–32^ Further, studies demonstrate no effect of intrapartum antibiotic therapy on time to culture positivity given use of culture media that neutralize antibiotics present in sampled blood.^33^ Time to positivity for most common neonatal EOS pathogens is <24 h, and the emergence of PCR-based diagnostics and mass spectroscopy are decreasing the time between the recognition of bacteremia, identification of pathogen and provision of antibiotic susceptibility information.^34^ The use of anaerobic culture may further enhance the ability to detect newborn bacteremia, particularly among preterm infants.^18,35^

## So why is so much antibiotic given to newborns for culture-negative sepsis?

Neonatal clinicians are currently armed with risk stratification strategies and increasingly refined means of identifying systemic infection. So what is the explanation for the AENEAS findings? It is possible that severity of neonatal illness, or markers of inflammation were used to make antibiotic decisions. Adult and pediatric studies have addressed the use of inflammatory biomarkers to rationalize antibiotic duration for suspected infection. For example, the PROGRESS (Procalcitonin-guided Antimicrobial Therapy to Reduce Long-Term Sequelae of Infections) trial enrolled 266 adults with Sepsis-3 criteria and demonstrated that PCT guided care reduced mortality and infection-associated adverse events, optimizing appropriate antibiotic use.^36^ However, biomarker use for neonatal EOS has been studied for decades and generally fails to improve diagnosis or antibiotic use in neonatal infection.^37,38^ Newer biomarkers such as micro RNAs and metabolomics are under exploration as complementary studies to add to blood cultures and clinical signs.^34^ Ultimately, one must ask: if treating culture-negative infection, what is being treated? What assumptions are made regarding potential pathogen, optimal antibiotic content and optimal duration of administration? There is not one easy path forward, but there are at least two areas where research may aid antibiotic stewardship for both culture-confirmed infection and the situations in which clinicians determine a need to extend antibiotics in the absence of confirmed bacteremia. First, most recommended durations of therapy among for culture-confirmed infection among newborns are based on expert opinion. Randomized control trials of antibiotic duration, accounting for pathogen and gestational age are needed to determine optimal duration and could inform culture-negative practice. Second, clinicians extend antibiotics when they start antibiotics. While significant progress has been made in quantifying EOS risk among term infants, large-scale studies to improve risk stratification among preterm infants could further identify infants who do not need antibiotics initiated. The AENEAS study findings clearly demonstrate that many neonatal clinicians consider blood cultures as necessary but not sufficient for the neonatal EOS diagnosis. In LMIC settings where blood culture is not widely available, clinical diagnosis of likely sepsis is critically important. But in settings where microbiologic technology is available and increasingly refined, the final area that needs research may be a qualitative one: to determine why in the face of evidence-based guidance, sophisticated diagnostics, and multiple studies (including AENEAS) that fail to find an advantage to extended, culture-negative antibiotic administration—so many clinicians opt to continue antibiotics once started. In the end, only a multicenter, randomized, placebo-controlled trial of extended antibiotic therapy using a clinician-driven definition of critical illness may satisfy those who adhere to the belief that more antibiotics are better. It is difficult to imagine such a study being successfully conducted with adequate statistic power among term infants. Perhaps the true lesson of the AENEAS study is that mortality was concentrated among infants with culture-confirmed infection, despite antibiotic therapy—and a detailed understanding of how the lowest antibiotic utilization centers achieve their practice would be a lesson to us all.
